# Atlanta Society of Medicine

**Published:** 1885-10

**Authors:** 


### Society Reports.

### ATLANTA SOCIETY OF MEDICINE.

Reported for The Atlanta Medical and Surgical Journal.

Stated Meeting, September 1, 1885.

Dr. J. D. Wilson in the chair.

Dr. W. S. Elkin brought before the Society a negro woman who was suffering from a large fibroid tumor of the uterus. The patient, Emma J., age 34 years, is tall, slender and rather emaciated. At the age of fifteen she began to menstruate and has always been regular in this respect. She is unmarried and was never pregnant. About six years ago she was accused by her friends and relatives of being pregnant, but she denied the unjust accusation, and sought the advice of a physician, who diagnosed the case as one of fibroid tumor of the uterus. Since then the tumor has steadily increased in size, but was attended with no special pain or inconvenience until two years ago, when there was more or less pain and hemorrhage during the menstrual flow. This trouble lasted only a few months, however, when the menses became natural again and void of any severe pain or loss of blood.

The tumor is globular, dense in structure, of stone-like hardness, and is doubtless sub-peritoneal in character. It is difficult to say if it is simple or multiple in form. We find indentations and nodules over its surface, and I am inclined to think it is multiple ; but these irregular bosses and excresences we feel here are often found on the parent tumor. The tumor is quite large, weighing perhaps forty or fifty pounds, and extends from the hollow of the sacrum, where it can be distinctly felt as a hard, immovable mass, to the diaphragm. It pushes the fundus downward and backward into Douglas' cul-de-sac. The os is high up, but can be felt between the tumor and the pubic symphysis. The sound measures three and a half inches. Although the tumor occupies the greater portion of the pelvic cavity, still, no difficulty has been experienced in the passage of the urine or in the movement of the bowels. As I said before, the tumor is steadily increasing in size, and on account of its great bulk is already beginning to interfere with the respiratory movements.

I have had the case under observation for more than eight months, and have been using palliative measures to check, if possible, the growth of the tumor and improve the general weakened condition of the patient. I have given ergot, cannabis indica, iodide of iron, potassium bromide and potassium iodide—such remedies that are known to lessen the flow of blood to the reproductive organs, and have used every means to prevent portal and pelvic congestion, but the size of the tumor still increases, not expanding so much anteriorly, yet gradually pushing itself upwards towards the floating ribs and diaphragm.

The broken down constitution of the patient has been built up by mineral and vegetable tonics. Having failed with my palliative remedies, I resorted to the method of radical treatment, as proposed by Prof. Hildebrandt, of Königsburg, which consisted in daily injections of ergot under the skin around the umbilicus. I employed the formula used by Dr. Ashhurst, which consisted of the fluid extract of ergot, diluted as follows:

| | |
|---|---|
| ℞—Fld. Ext. Ergotæ | f℥iss. |
| Glycerinæ | f℥i. |
| Aquæ | f℥ii. |

Twenty drops of this solution, containing about seven drops of ergot, were injected daily (except during menstrual flow) in the sub-umbilical region. To avoid abscesses the injections were made deep, the nozzle of the syringe being carried down to the muscular parietes. Accurate measurements of the size of the tumor were taken, and this treatment kept up for nearly two months with no diminution in the substance of the tumor. No abscesses were produced by the sub-cutaneous injections, but the pain caused by the physiological effect of the drug was so severe and depressing to the patient that I was compelled to abandon this treatment.

I am inclined to believe that the only recourse now left us is a removal by laparotomy of the pedunculated growths or the wholesale extirpation of the womb and its appendages. It is true, that the mortality in these operations is great, yet, I am convinced that the mechanical obstruction caused by the presence of this large growth will soon kill the patient.

Dr. Hardon thought that an operation in this case perfectly justifiable, and he would feel no hesitancy in performing it.

Dr. Wile agreed with Dr. Hardon as to the advisability of operating, on the ground that the tumor would prove fatal to the patient through mechanical obstruction, and that soon, as evidenced by its present interference with the respiratory organs.

Dr. Wilson stated that in almost every case he had seen operated on, where the tumor was as large as the one just examined, death followed, and he did not believe we could hope for good results in this instance. He thought that owing to the immobility of the tumor in the pelvic cavity, we would find adhesions along the course of the rectum, which would greatly augment the danger if an operation was performed. He didn't think he could conscientiously perform the operation in this case.

Dr. Howell favored the further continuance of palliative means, and thought an operation would prove fatal, and was therefore not justifiable.

Dr. Elkin closed the discussion by saying he believed the operation should be performed, and as the patient's condition was at present somewhat improved, he deemed it proper not to delay the procedure too long. He did not think that the size of the tumor was sufficient grounds for not operating. He had seen favorable results where the tumor was larger in appearance than this. He said the gravity of the operation would depend principally upon the exact character and position of the tumor, and the number of adhesions encountered during the operation.

Dr. Virgil O. Hardon reported

A CASE OF VASCULAR TUMOR OF THE MEATUS URINARIUS IN A CHILD THREE YEARS OF AGE.

A. B., female, white, aged three years, was brought to me July 14 by her father, who stated that a little over a year ago he began to notice that she frequently rubbed her genitals with her hands, and that she would lie on the floor in such a position as to produce friction of the genitals against the carpet. She also complained of pain and frequency of micturition. No other symptoms attracted attention. She has always been well. Her appetite is good, bowels regular, sleep natural, except when broken by the desire to urinate. There is no incontinence of urine.

She has the appearance of a healthy, robust, well nourished child. Pulse 72, strong and regular, temperature normal. Examination showed hyperæmia of the mucous membrane, covering the labia and surrounding the meatus. At the lower margin of the meatus was a vascular tumor about the size of a grain of wheat, overlapping the orifice so as partially to occlude it. There was no hyperæsthesia of the tumor nor of the surrounding mucous membrane. It bled readily upon being touched.

July 15.—Patient was anæsthetized with Squibbs' ether, about two fluid ounces being used. With curved scissors the growth was removed. Some bleeding followed, which was checked by the application of Monsell's solution. No dressing was applied, and the patient was allowed to ride home in the horse cars after the operation. A solution of sulphate of zinc was ordered to be applied to the inflamed mucous membrane.

July 24.—Patient still has some irritation of the urethra, shown by pain and frequency of micturition. Ordered a mixture of buchu, pareira brava and acetate of potash.

Aug. 25.—Father reports that all the symptoms of urethral irritation have disappeared.

A CASE OF VERRUCA VULGARIS.

Dr. Wile reported the case not so much on account of any special peculiarity connected with it, but merely as a matter of diagnosis.

A young woman twenty-eight years of age exhibited a flat, splitpea sized, slightly elevated, sharply defined lesion with uneven surface situated on the upper part of the right cheek. Just beside the lesion was a white pea-sized cicatrix, the result of the removal of a lesion similar to the present one, and which had existed one year ago. It was pronounced cancer, and excision was resorted to.

The surface of the surrounding skin was the seat of irritation, some seborrhoeic discharge, and slight burning pain, all of which was due to irritating applications that were constantly being made to the lesion, stimulating it to grow, and setting up a dermatitis in the surrounding healthy skin. Applications of hot water were ordered twice daily—followed by a mild ointment.

℞—Sulphuris præcipitati.................... ʒss.

Ung. Aquæ Rosæ...................... ℥i.

M. ft. ungt.

Sig. To be rubbed in gently twice daily.

The third day after the treatment all irritation, discharge and pain ceased, and there remained only the wart, which the patient objected to having removed at present, she being greatly relieved in mind on hearing of the benign character of the lesion. The wart is of the common variety, and its removal at any time will be an easy matter.

